# Developing a fully integrated tobacco curriculum in medical colleges in India

**DOI:** 10.1186/s12909-015-0369-3

**Published:** 2015-05-20

**Authors:** T. R. Yamini, Mark Nichter, Mimi Nichter, P. Sairu, S. Aswathy, K. Leelamoni, B. Unnikrishnan, Prasanna Mithra P., Rekha Thapar, S. R. Basha, A. K. Jayasree, T. R. Mayamol, Myra Muramoto, G. K Mini, K. R. Thankappan

**Affiliations:** 1 Achutha Menon Centre for Health Science Studies, Sree Chitra Tirunal Institute for Medical Science and Technology, Thiruvananthapuram, Kerala India; 2 University of Arizona, School of Anthropology, 85721 Tucson, AZ USA; 3 Department of Family and Community Medicine, University of Arizona, Tucson, AZ USA; 4 Department of Community Medicine, T.D. Medical College, Alappuzha, Kerala India; 5 Department of Community Medicine, Amrita Institute of Medical Sciences, Kochi, Kerala India; 6 Department of Community Medicine, Kasturba Medical College, Manipal University, Mangalore, Karnataka India; 7 Department of Community Medicine, Bangalore Medical College and Research Institute, Bangalore, Karnataka India; 8 Department of Community Medicine, Academy of Medical Sciences, Pariyaram, Kannur, Kerala India

**Keywords:** Tobacco curriculum, India, Tobacco cessation

## Abstract

**Background:**

This paper describes a pioneering effort to introduce tobacco cessation into India’s undergraduate medical college curriculum. This is the first ever attempt to fully integrate tobacco control across all years of medical college in any low and middle income country. The development, pretesting, and piloting of an innovative modular tobacco curriculum are discussed as well as challenges that face implementation and steps taken to address them and to advocate for adoption by the Medical Council of India.

**Methods:**

In-depth interviews were conducted with administrators and faculty in five medical colleges to determine interest in and willingness to fully integrate smoking cessation into the college curriculum. Current curriculum was reviewed for present exposure to information about tobacco and cessation skill training. A modular tobacco curriculum was developed, pretested, modified, piloted, and evaluated by faculty and students. Qualitative research was conducted to identify challenges to future curriculum implementation.

**Results:**

Fifteen modules were successfully developed focusing on the public health importance of tobacco control, the relationship between tobacco and specific organ systems, diseases related to smoking and chewing tobacco, and the impact of tobacco on medication effectiveness. Culturally sensitive illness specific cessation training videos were developed. Faculty and students positively evaluated the curriculum as increasing their competency to support cessation during illness as a teachable moment. Students conducted illness centered cessation interviews with patients as a mandated part of their coursework. Systemic challenges to implementing the curriculum were identified and addressed.

**Conclusions:**

A fully integrated tobacco curriculum for medical colleges was piloted in 5 colleges and is now freely available online. The curriculum has been adopted by the state of Kerala as a first step to gaining Medical Council of India review and possible recognition.

## Background

### Introduction and aims of this paper

India is the second largest producer and consumer of tobacco in the world with 48 % of men and 20 % of women using some form of tobacco [1]. Among the 275 million tobacco users in the country, approximately 25 % of men and 3 % of women are smokers [1]. Tobacco-related mortality in India is among the highest in the world, with an estimated one million deaths annually attributable to smoking. Of those who die from smoking-related illnesses, 70 % are between the ages of 30 and 69. Due to continued population growth, the number of deaths in this age group is rising by about 3 % per year [2].

Reducing tobacco-related morbidity and mortality is a global health priority that requires both tobacco prevention and cessation efforts. To date, far more attention has been focused on prevention than cessation in low and middle income countries (LMICs). Research globally indicates that an increase of just 1 % in long-term cessation rates can positively impact public health [3]. In India, a 1 % decrease in tobacco use would result in 7.8 million fewer tobacco users [4]. Making tobacco cessation a normative part of all clinical practice is the only way to substantially reduce tobacco-related morbidity and mortality in the short term [5]. In addition, illness is a teachable moment and doctors need to routinely ask patients about their tobacco use and advise them to quit after informing them about the harms of tobacco [6].

Three notable challenges face those attempting to introduce cessation training to the next generation of doctors in LMICs. First, it is necessary to raise the consciousness of medical college faculty about the many harms of tobacco use beyond cancer. Second, medical students need to be socialized to talk to patients about their tobacco use. And third, students need to be taught culturally appropriate ways of counseling patients who are ambivalent about giving up tobacco use or have not yet considered doing so.

These three challenges were taken up in Project Quit Tobacco International (QTI) by a multidisciplinary team of tobacco researchers from the University of Arizona, Sree Chitra Tirunal Institute of Medical Science and Technology in Kerala, India, and Gajah Mada University, Indonesia. This research team has spent the last ten years developing tobacco cessation curricula for medical colleges in India and Indonesia, adapting evidence-based cessation approaches to fit local cultures, and promoting community-based smoke free home campaigns as a means of reducing women and children’s exposure to second-hand smoke. During the formative research stage of Project QTI (2002–2007), qualitative and quantitative research was conducted to ascertain the extent to which doctors in India and Indonesia engaged in delivering cessation messages to patients, and inclusion of information about the harms of tobacco and cessation skills in the medical college curriculum [7]. We also collected data on laypersons’ perceptions of the harm of smoking at low to moderate levels both among those who were relatively healthy and those afflicted with acute and chronic diseases [8]. In this paper, we briefly highlight a few key findings of this research that served as the impetus for a second stage of intervention research (2007–2014), which entailed the development of a novel, fully integrated tobacco education curriculum in medical colleges [9]. We then describe the pretesting and piloting of this medical curriculum in five medical colleges in South India, report on how the curriculum was received, and discuss next steps being taken to implement the curriculum elsewhere in the country in a flexible manner.

The main focus of the project’s cessation activities in India has been smoked tobacco although prevalence of smokeless tobacco is high. We chose to focus on cigarette and beedie smoking because it is associated with a wider range of health problems than smokeless tobacco including the health hazards of second hand smoke. The harms of chew tobacco are covered in the QTI curriculum, but chew tobacco was not the focus of cessation training. Expanding cessation training to include chew tobacco is a high priority future agenda.

### Smoking cessation in India: an overview

According to the Global Adult Tobacco Survey India (GATS 2009–2010), slightly less than half of all smokers in India and less than a third of all smokeless tobacco users have been asked by their health care providers to quit at any point in time [1]. Formative research conducted by Project QTI in Kerala found that during medical consultations, the vast majority of patients in India are not asked if they smoke nor advised to quit by health care providers [10]. Results reveal that many health care practitioners in the public and private sector believed that smoking a few cigarettes a day was relatively harmless. In a survey of over 300 doctors, about one third reported that smoking only becomes harmful when the number of cigarettes smoked per day is six or more [11]. Formative research also revealed that cessation advice was not seen as an important part of TB or diabetes treatment, despite the fact that tobacco is related to TB relapse and serious diabetes complications [12, 13].

For example, a QTI survey of 440 male diabetes patients attending clinics in Kerala revealed that 44 % had smoked one week before diagnosis and 23 % were current smokers. One hundred diabetes patients who were recent or current smokers were interviewed. Two thirds of these diabetic smokers did not think smoking affected their diabetes and most thought smoking six cigarettes or less a day was relatively harmless [13]. While 75 % had been told to quit or cut down smoking by their doctor, most were told to do so only at the time of diagnosis. The quit messages they received from doctors were general and not disease specific and were often interpreted to apply to heavy smoking.

In recognition of the importance of smoking cessation, 13 smoking cessation clinics were started in 2002 by India’s Ministry of Health and Family Welfare with the support of the World Health Organization, toward an end of testing cessation strategies for smokers and smokeless tobacco users. This number has increased slightly to 19 clinics as of 2014, but all clinics are urban based [10]. Moreover, it has not been within the scope of their clinic-based activities to develop a cessation curriculum for India’s medical colleges. The number of cessation clinics currently operating in India is miniscule in relation to the country’s needs and doctors clearly need to be trained to encourage and support cessation [14, 15].

### Tobacco education in Indian medical colleges

A medical curriculum audit carried out by QTI researchers found that medical colleges devoted little time to tobacco as a disease risk factor. Calling attention to the negative effects of tobacco on organ systems, disease processes, or medicine effectiveness was left to individual faculty members and was not a mandated part of the formal medical curriculum. Cessation skills were not covered in medical college classes nor demonstrated on the wards or during community medicine postings.

Interviews with patients revealed that when doctors did provide cessation messages, these messages were commonly interpreted to mean “do not smoke when ill, and when you are too sick to smoke.” Messages not to smoke during illness episodes unrelated to the lungs were often interpreted as general advice, not specific advice for their health condition. Exit interviews with patients found that patients often doubted the relationship between tobacco and their health condition [8]. Those afflicted with diabetes, hypertension, back pain, earache, gastritis, and a wide range of other illnesses questioned how smoking could possibly affect their health condition and requested an explanation of the connection between smoking and their immediate health complaints. This was found to be the case irrespective of the educational level of the patient.

Among doctors, QTI researchers found low levels of confidence for counseling patients for cessation beyond giving general and largely paternalistic advice to quit [16]. Many doctors voiced a sense of frustration regarding what they could say to patients when their advice to quit went unheeded. Observations of doctors who did give quit advice to patients revealed that many did so by scolding patients that tobacco was a dirty habit that was bad for health.

Interviews with medical students identified a general sense that if a subject was important, it was raised in many lectures during their four and a half years in medical college, and covered by exam questions. Passing comments about the harms of tobacco in an occasional lecture made little impression. Moreover, smoking inside the medical campus, especially by faculty and senior students, gave some students the idea that smoking could not really be that bad a health problem if one did not smoke in excess. On the other hand, campus wide surveys revealed strong support for medical campuses to become smoke free and for more attention to be allocated to tobacco in the medical curriculum [16]. Many students voiced personal concern about family members who smoked.

These research findings led us to conclude that it was necessary to develop a much different type of tobacco curriculum for medical colleges than that reported elsewhere [17, 18]. We saw a need to fully integrate tobacco education into all semesters of the medical curriculum as a means to tie illness and organ-specific information to cessation. Only in this way would young doctors be trained to establish the relevance of quit advice during medical consultations as teachable moments. Tobacco education needed to be mainstreamed in medical education and cessation needed to be seen as a central part of care management rather than being sidelined as an add-on topic.

The three primary objectives for integrating tobacco in medical education were: 1) to raise the consciousness of medical students about the systemic harms of tobacco and their link to multiple health problems, and 2) to provide students with the means to establish the relevance of their cessation advise to patients during illness as a teachable moment. Formative research carried out by QTI found that patients being given cessation advice wanted doctors to explain to them how smoking was related to their specific health condition [8, 9]. Educational modules were designed to provide students with basic epidemiological data on smoking, an understanding of the mechanisms by which tobacco caused or exacerbated a full range of health problems, and the impact of smoking on medicine effectiveness. The third objective was to provide medical students with both basic cessation skills (5 As) and more advanced cessation skills (5 As plus), which incorporated establishing relevance between advice and disease specific relevance. Establishing relevance is the first of the 5 Rs employed to counsel patients who have some reluctance to quit smoking. Formative research found that most patients are reluctant to quit, and had little comprehension that smoking was bad for their specific health condition, unless it was cancer.

## Methods

Five lead medical colleges were identified as sites in which to develop and test tobacco curriculum for undergraduate medical students. These colleges were chosen to represent a range of public and private institutions. Medical colleges selected were located in both urban and rural areas of two states in South India Kerala, and Karnataka. Ethical clearance for the project was given by the Sree Chitra Tirunal Institute for Medical Sciences and Technology in Trivandrum, Kerala.

Following preliminary meetings with college administrators, it was determined that the project should be housed in Departments of Community Medicine that were responsible for coordinating preventive and promotive research activities at their respective schools. One faculty member from Community Medicine was appointed at each college to coordinate tobacco curriculum activities and to work closely with the head of the department who had overall responsibility for the program. The head of each department selected the faculty member(s) who delivered the tobacco modules to the students.

We first examined the existing medical curriculum to see where and how tobacco was currently being taught. India has a standardized medical curriculum determined by the Medical Council of India (MCI) with guidelines, objectives, and specific topics specified for both undergraduate and postgraduate education. The structure and timing of implementation is flexible and varies widely across programs. Curriculum review showed that tobacco-related information was confined to only four courses in the four and a half years of medical college—pediatrics, surgery, respiratory medicine, and community medicine. However, even in these courses, coverage was minimal.

Working with faculty from each medical college, QTI researchers quickly realized that a “one size fits all” approach to tobacco education would not work in India. The curricula would need to be sufficiently flexible to accommodate differences between institutions. Curriculum mapping helped us better understand the semester in which the tobacco-related modules we were developing could most appropriately be introduced at each partner school and which faculty should be responsible for material to be covered.

From the onset, we recognized systemic challenges. First, we had to become familiar with students’ levels of comprehension of science in different years of medical education and across medical colleges. The information contained in the teaching modules clearly had to be tailored to students’ emerging level of understanding. Second, some semesters of the medical curriculum were already quite full, leaving little room for the introduction of new classes. Core material had to be integrated into existing lectures in a seamless manner. Third, high staff turnover demanded that teaching materials be well packaged and easy to pass on from one faculty to the next without much explanation or preparation time.

### Module development

To create the curriculum, a transdisciplinary team of QTI researchers from India, Indonesia, and the U.S. reviewed evidence-based findings from peer-reviewed journals to identify key facts to be incorporated in the tobacco modules. This process took place over an 18 month period of time. The main component of modules initially developed were PowerPoint presentations of 20–30 slides focused on the impact of tobacco on specific organ systems, diseases related to tobacco use, medication effectiveness, and the need to explain the importance of quitting smoking before an illness becomes much worse. Each slide was developed with extensive speaker notes. Faculty was also provided key articles from the expert literature from around the world [19].

Initially, ten distinct modules were developed on the effects of tobacco on the cardiovascular system, respiratory system, gastrointestinal system, nervous system, reproductive system, musculoskeletal pain, endocrine problems, mental health, and tobacco and neoplasia. An overview module on tobacco as a global health priority, tobacco control strategies, and the state of tobacco consumption in India was developed for use in community medicine and a module on basic cessation counseling skills (the 5 As: ask, advise, assess, assist, arrange), was developed for a class on basic medical practice [5, 20].

In addition to the PowerPoint presentations, a module also included fact sheets, case scenarios, and short clinical videos demonstrating the 5 As for patients with different types of health problems. Fact sheets for each module were provided to students as a study guide and faculty were provided sample exam questions. Illness specific brief cessation counseling was modeled in videos of 7–10 min duration and clinical case scenarios were provided for use in seminars and role play activities. Sample examination questions were included for each module because if a topic is not included in examinations in India, it is not given much importance by the students.

### Module pretesting

After completion of 10 modules, a curriculum specialist from Project QTI visited each of the colleges, where a group of five faculty members from diverse faculties were tasked with critically reviewing specific modules. Initial feedback indicated that faculty members were impressed with the breadth and depth of the PowerPoint presentations. Modules reviewed tobacco as a risk factor for particular types of pathology, provided statistics on mortality and morbidity, explained the mechanisms behind pathology, and placed emphasis on positive health outcomes of quitting smoking/chewing tobacco. Some faculty did, however, express concern about module length given the amount of time they could allocate to the subject of tobacco in a one-hour class.

Differences of opinion were expressed with respect to module length. Some faculty preferred that the lecture be shortened and only highlight a few core issues so that it could be inserted into existing lectures. Other faculty asked for educational resources that could be linked together to form longer lectures. Faculty also pointed out that some of the language utilized might be difficult for students and suggested simplifications and corrections for slides that contained too much information. Faculty particularly appreciated the speaker’s notes, stating this gave them the confidence to lecture on a new subject.

At each college, we elicited feedback for each mini lecture from groups (comprised of 10 students per group) who sat through a presentation by faculty members. These students were typically in their 2^nd^ to 4^th^ year, depending on when the focal module was covered at their medical college. Students were given a short evaluation form which contained close-ended and open-ended questions. Most students found the presentations interesting and stated that they provided them a heightened understanding of the role of tobacco in disease processes. Students indicated where slides needed to be simplified and requested that more visuals be included in the modules.

### Developing the “Lego” approach

Based on the feedback received, we modified the modules substantially, subdividing the long PowerPoint presentations into two to five mini lectures to provide flexibility to a faculty member who could either insert these smaller presentations (comprised of four to seven core slides) into an existing lecture, or string them together to form a single lecture, if time permitted (Table 1). We further divided mini lectures into core message slides, country specific data slides, and optional slides so that faculty could choose how much they would present on a particular tobacco-related topic. Language was simplified to the extent possible, and additional visuals were added. The specific objectives for each mini lecture in the tobacco curriculum for medical colleges is available on the Project QTI website: www.quittobaccointernational.org.

**Table 1:** Modules and mini lectures

No:	Module title	Power point presentations: mini lectures (ML)	Semester given
**1**	Tobacco issues in basic medical practice and professionalism	ML-1 Burden of Tobacco	1
		ML-2 Tobacco as a risky behavior for patients and the family	1
		ML-3 Role of doctors in tobacco control, including 5As	3
**2**	Tobacco and Community Medicine and Public Health	ML-1 Burden of tobacco and global tobacco control	1
		ML-2 Tobacco and occupational health	1
		ML-3 Hazard of passive smoking and smoke free environment	1
		ML-4 Cessation strategies and social support	1
		ML-5 Tobacco impact on the family and economy	1
**3**	Tobacco and the musculoskeletal system	ML-1 Nicotine effects on pain.	3
		ML-2 Tobacco and back pain and osteoporosis	6
**4**	Tobacco and the endocrine system	ML-1 Tobacco and diabetes	3
		ML-2 Tobacco and other metabolic disorders (hyperlipidemia, obesity)	3
**5**	Tobacco effects on the cardiovascular system	ML-1 Epidemiology of CVD and tobacco	3
		ML-2 Tobacco as CVD risk factors and the mechanism	3
		ML-3 Important of cessation in patients with CVD	3
**6**	Tobacco and the gastrointestinal system	ML-1 Tobacco and oral diseases	1
		ML-2 Tobacco and common abdominal disorders	6
**7**	Tobacco and the reproductive system	ML-1 Tobacco effects on pregnancy	3
		ML-2 Tobacco effects on fertility	3
		ML-3 Passive smoking and pregnancy	3
**8**	Tobacco effects and neoplasia	ML-1 Tobacco and carcinogenesis	4
		ML-2 Tobacco and respiratory tract cancer	4
		ML-3 Tobacco and digestive cancer	4
		ML-4 Tobacco and urogenital cancer	4
		ML-5 Tobacco cessation as an important part of cancer management	4
**9**	Tobacco and the nervous system	ML-1 Tobacco and stroke	4
		ML-2 Tobacco and pain	4
		ML-3 Nicotine effects on neurodegenerative diseases	4
**10**	Tobacco effects on the respiratory system	ML-1 Tobacco and TB	6
		ML-2 Tobacco and COPD	6
		ML-3 Tobacco and asthma	6
**11**	Tobacco and mental health	ML-1 Nicotine addiction	6
		ML-2 Tobacco and depression and anxiety	6
		ML-3 Tobacco and schizophrenia	6
**12**	Tobacco and the sensory organs	ML-1 Tobacco and the aging eye (cataract and age related macular degeneration)	7
		ML-2 Tobacco and skin (aging and wound healing)	6
**13**	Tobacco and children’s health	ML-1Tobacco effects on respiratory health in children	5
		ML-2 Other tobacco effects on children’s health	5
**14**	Tobacco and the uropoetic system	ML-1 Tobacco effect and erectile dysfunction	6
**15**	Tobacco and adolescents	ML-1 Tobacco use among adolescents	1
		ML-2 Effect of tobacco use on adolescent growth and development	1
		ML-3 Risk factors for tobacco initiation	1
		ML-4 Tobacco advertising and marketing to youth	1
		ML-5 Tobacco prevention and cessation for adolescents	1

Note: For additional information on the specific objectives for each module, refer to the tobacco curriculum for medical colleges on the Project QTI website: www.quittobaccointernational.org

**Fig. 1:**
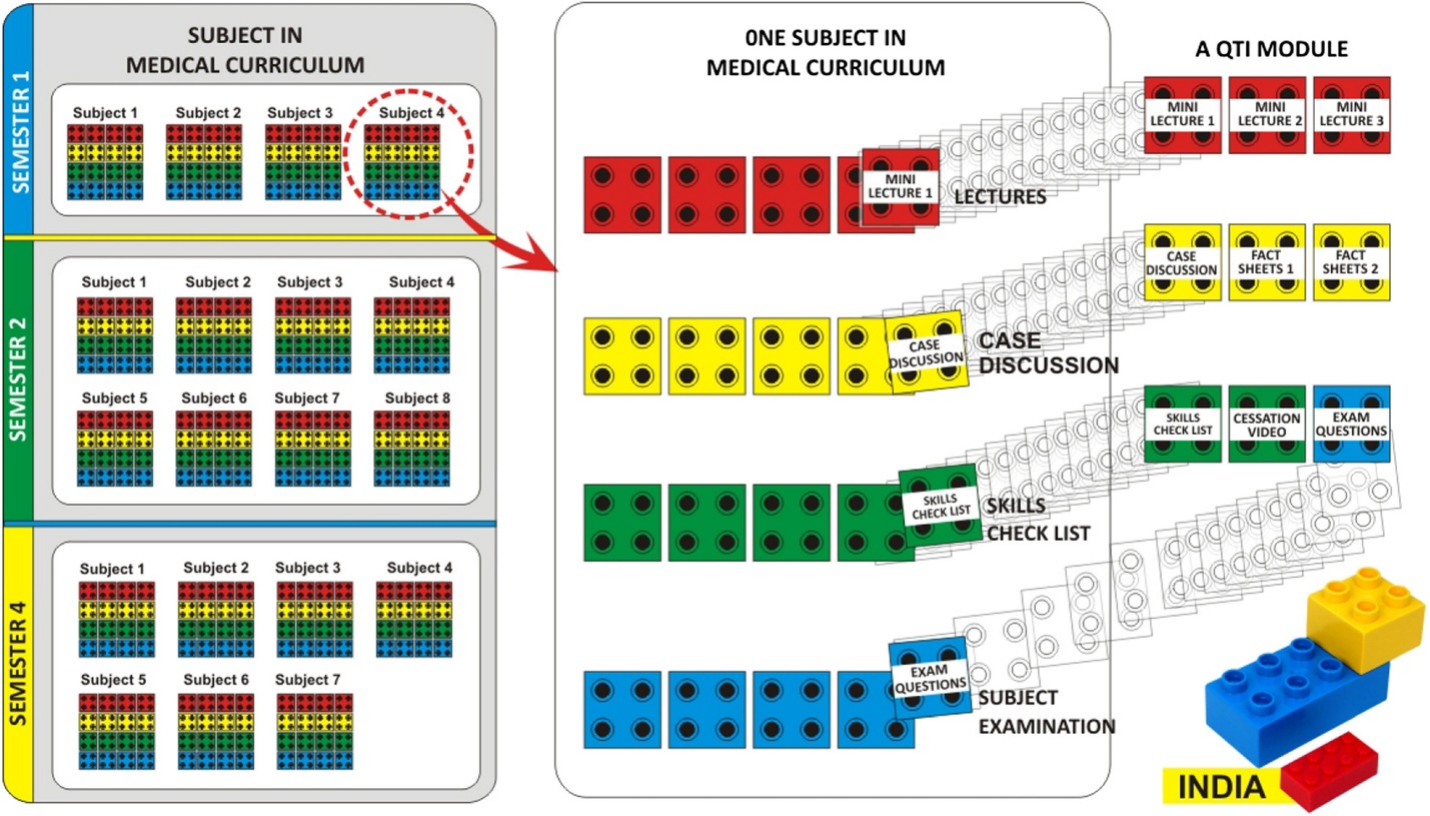
Building a tobacco curriculum through a “Lego” approach

This string of mini lectures—the modular “Lego” approach—provided faculty with standardized curriculum building blocks assembled into easy to use modules comprised of five parts: mini lecture, fact sheets, cessation videos, case scenarios, and exam questions (Fig. 1). Like Legos, the pieces of the module can fit together in a variety of ways to form different configurations. We encouraged each medical college to experiment with how best to assemble the Legos to fit the needs of their existing curriculum. For example, faculty could choose whether to present one or more mini lectures in a single class or whether to discuss a case scenario or show a brief intervention video modeling cessation skills in a small group setting.

## Results

### Piloting of modules

As a means of ensuring that the material contained in the modules was of acceptable quality, we had faculty members from each sub specialty from one or more colleges review and then teach the module. During the year of pilot testing (2011–2012), faculty members from appropriate departments taught classes using their department-allotted lecture hours or when necessary, during Community Medicine hours dedicated to that subject area. In some cases, topics covered in the QTI curriculum did not easily fit into an undergraduate medical curriculum. For example, radiation oncology is not taught to undergraduate students in some colleges and therefore, respiratory medicine took up the mini lecture on lung cancer using their hours or Community Medicine hours.

During piloting of the curriculum, 3500 students were exposed to some, if not all, of the mini lectures contained in a revised set of 15 modules (see Table 1), their exposure determined by students’ year in medical college. An evaluation of the curriculum by 917 students from the five medical colleges found that 91 % rated the modules as highly informative, and 75 % reported they now felt confident to engage in cessation counseling. Of these 917, only some had actually had the experience of counseling patients, a subsample that we report on below.

### Implementation

Colleges were asked to implement the curriculum as they saw fit. As anticipated, different colleges implemented the curriculum in different ways. In some colleges, modules such as tobacco and child health were integrated into existing classes on pediatrics by a faculty member in the department. Similarly, modules such as tobacco and cardiovascular disease or tobacco and the gastrointenstinal system were taught by faculty in the department of general medicine (i.e., internal medicine) or general surgery.

A second implementation style adopted was tobacco modules being taught using the hours reserved for Community Medicine lectures related to a topical area. In all partner medical colleges, Community Medicine is allocated hours in medical specialties across the first three and a half years. When a course already had a very full curriculum and the faculty member had no time to introduce new material into regular lectures, the lectures were given using Community Medicine hours. For example, at one college a Community Medicine faculty member taught a class on tobacco and mental health, as the department of psychiatry faculty were unable to fit tobacco into their existing class schedule.

#### Teaching cessation skills

The primary reason for exposing medical students to illness specific knowledge about the harm of tobacco was to support a patient-centered approach to smoking cessation. Students were trained to establish the relevance of quit advice by drawing a link between smoking and the health problem suffered by the patient at the present and a likely health scenario in the future. We found that without a student linking quit advice to a patient’s illness, patients were not interested in talking to them about tobacco; many patients had the feeling that general advice about smoking was simply part of a student’s training and not specific to their condition. Once relevance was established, students were taught basic cessation related communication and motivational interviewing skills.

We first established training capacity in these skills by holding workshops for faculty members who would serve as trainers in their college. Each school trained at least two faculty members. Cessation training introduced faculty to the 5 As “plus” (i.e., establishing illness specific relevance); assessing patient’s stages of readiness to quit [20, 21] and matching advice to readiness; the 5 Rs (relevance, risk reward, roadblocks, repetition) [5] as a way of motivating patients reluctant to quit; and culturally sensitive ways of dealing with 1) physical barriers to quitting (withdrawal), 2) psychological barriers (craving, use of smoking as self medication for stress), and 3) social challenges like dealing with peer influence and/or learning how to refuse offers of cigarettes/beedies.

Following two-day training workshops, faculty members were required to conduct 15 cessation interventions with patients and to fill out logs documenting the skills they employed during counseling and short notes on how patients responded. These logs were then submitted to Project QTI staff and formed the basis of an hour long oral examination that both tested faculty members’ knowledge and application of skills and served as a one-on-one consultation where problems encountered could be discussed. Faculty members completing this test received accreditation in basic cessation communication skills, awarded by the University of Arizona and Sree Chitra Tirunal Institute for Medical Science and Technology.

Medical students were initially exposed to the 5 As plus approach in either their first or second year. In subsequent years, they were presented examples of brief cessation interventions employing the 5 As plus approach modeled in illness specific brief intervention (BI) videos. In total, Project QTI produced fourteen videos modeling doctor-patient cessation skills in either English or Malayalam languages. Videos were also made available to students on the QTI website so they could refer to them at their leisure. When students began to see patients (usually in the second year of medical college), they were reintroduced to the 5 As and gradually taught additional cessation skills. These skills enable them to support quit attempts, interact with patients ambivalent about quitting, and deal with physical, psychological, and social barriers to quitting. Thus, students initially began using the 5As while doing role play activities and then gained real life experience through interactions with patients on the ward. To standardize the training, a 90 min multi-segment training video was developed that presents the same package of cessation skills taught to faculty trainers. Depending on the college, students in the 3^rd^, 4^th^, or 7^th^ semester were asked to conduct and document five cessation counseling attempts with patients. These five BI logs have become part of their Community Medicine activity record book submitted for evaluation. In some colleges, cases were discussed in groups of five students, while in other colleges skills were further assessed through role play or Objective Structured Clinical Examination.

Three hundred and forty medical students distributed across the five medical colleges who finished cessation training and completed five patient counseling sessions took part in an evaluation of the training program. On an anonymous survey, 90 % reported that they now felt confident to counsel patients to quit smoking. Seventy-nine percent stated that they were both routinely asking patients about smoking and counseling them to quit. Eighty percent had viewed a mean of five training videos modeling illness specific counseling skills. Of these students, 94 % stated that watching the videos was very helpful above and beyond lectures on the subject. Seventy-eight percent of the 340 students stated they had recently tailored cessation counseling to a patient’s illness. Notably, the list of health problems specified ranged well beyond respiratory illnesses and types of cancer to diabetes, heart conditions, back pain, and gastric complaints. Eighty-six percent strongly agreed with the statement that a patient’s chance of quitting smoking was far greater if a doctor advised them to quit. Ninety-nine percent agreed with the statement that it was important for all medical students to be trained in smoking cessation. Notably, 57 % had already counseled a family member about quitting smoking and another 19 % stated that they planned to in the near future. Previous research in India has shown that incorporating 5As interventions in doctor-patient interactions increases the patients’ satisfaction with physicians’ delivered counseling services and aids in demand generation for cessation services [22].

## Discussion

### Challenges facing future implementation

Although the curriculum had been well received by faculties at the five medical colleges, coordinating curriculum implementation has been labor intensive as faculty from clinical faculties have to be followed up by coordinators to see if, when, and how they have taught tobacco modules. Until such time that tobacco is more fully integrated into the curriculum and approved by the MCI, there will be a need for ongoing advocacy to include tobacco as an additional but high priority topic.

To gain traction, logistic challenges will have to be faced. In most medical colleges, there is a very high staff turnover, about 20–40 % per year. Further, faculty members are often assigned different subjects from year to year. What this means is that a faculty member who has taught a module one year might well be posted elsewhere the following year, requiring the tobacco curriculum coordinator to find a new faculty member to teach the module. At some of the colleges, the heads of various departments were willing to identify a suitable faculty member, but at other schools Community Medicine coordinators found it more time effective to take the classes themselves using their own hours allocated to that subject. The general consensus among the five coordinators was that the ideal lay somewhere in the middle: involving as many interested faculty as possible while at the same time having Community Medicine faculty serve as a backup who could teach if coordination became burdensome. It was recognized that it was most advantageous for faculty across disciplines to teach about tobacco, as this would result in students viewing the topic as more important.

The five departments of community medicine participating in this project enthusiastically took up a leadership role in smoking cessation as they recognized the importance of the topic as well as the fact that it increased the status of their department in the medical college. In recent years, there has been a growing recognition of the importance of lifestyle factors as contributors to both communicable and non-communicable diseases. Smoking cessation provides departments of community medicine with an example of a lifestyle factor that is relevant to all subspecialty areas of medicine. The role of community medicine in tobacco control establishes their discipline’s rising importance in the medical field and has led to greater interaction with other clinical departments.

A new opportunity for community medicine to introduce tobacco has been afforded through an integrated teaching program being introduced in some medical colleges in Kerala and Karnataka. In this scheme, three or four departments collaborate in addressing a common topic. For example, at one college, community medicine, respiratory medicine, pathology, and general medicine joined together to teach about lung cancer, allowing the topic to be taught in an integrated manner. Similarly, microbiology, community medicine and pediatrics joined together to teach about acute respiratory infection. Opportunities like this related to shifts in pedagogy can be maximized for the introduction of tobacco in problem-based learning.

### Next steps toward the goal of integration

One of QTI’s overall goals has been advocacy, to provide the MCI with a compelling evidence-based case study illustrating how smoking cessation may be integrated into the country’s undergraduate medical college curriculum. A first step toward the ambitious goal of MCI curriculum approval has been realized in Kerala state. The Kerala University of Health Sciences, which approves all undergraduate medical curriculum at the state level, was approached by Project QTI. After review of the five medical college pilot study, the Vice Chancellor and Dean of Research supported the adoption of the QTI curriculum and called for a meeting of all the medical colleges in the state. Representatives of 12 of the 25 medical colleges in Kerala attended a first meeting in March 2014 and were asked to assemble a working group of eight faculty members at each college to consider how best to implement the modules and cessation training. First generation QTI partner colleges have stepped forward to offer advice on how and when to implement the curriculum. Three of the first generation QTI partner medical colleges have also become mentors to second generation colleges, assisting them train faculty and adopt the QTI curriculum (two colleges in Kerala and two in Karnataka). Steps are also being taken to involve dental schools in researching best practices for promoting smokeless tobacco cessation.

## Conclusions

This paper describes a pioneering attempt to introduce a fully integrated tobacco curriculum in Indian medical colleges. The curriculum trains medical students to use patient consultations as teachable moments where they have the opportunity to establish the relevance of quit smoking advice by calling attention to how smoking affects a patient’s current health problem and is likely to compromise their health in the future if they do not quit. This patient centered approach to smoking cessation points to their current illness as a warning sign and wake up call.

The tobacco curriculum developed is available on the QTI website (www.quittobaccointernational.org). It is the product of collaborative participatory research and is not a simple adaptation of a curriculum transferred from the West. The curriculum is culturally sensitive, evidence-based, and developed, pretested, and piloted in real world Indian medical college conditions. Logistical challenges have been identified and addressed. The curriculum is flexible enough to meet the challenges of shifts in India’s changing medical curriculum and high staff turnover and affords Indian faculty the opportunity to be creative in integrating the Lego like components of the modules supplied as they see fit.

This project was undertaken towards the end of developing and testing a model tobacco curriculum for medical colleges in India. Our hope is that it will inform the MCI in its future deliberations on how to integrate tobacco more fully in all of India’s medical colleges. In light of the impending non-communicable disease epidemic in India, where tobacco has a pivotal role, including tobacco in the curriculum is urgently needed.

## Abbreviations

LMIC: Low and middle income countries
QTI: Quit Tobacco India
GATS: Global Adult Tobacco Survey
MCI: Medical Council of India
5As: Ask, advise, assess, assist, arrange
5As plus: Using the 5As while conducting illness specific counseling
5Rs: Relevance, risk, reward, roadblocks, repetition
BI: Brief intervention
